# Glyceryl Tribenzoate: A Flavoring Ingredient, Inhibits the Adoptive Transfer of Experimental Allergic Encephalomyelitis via TGF-β: Implications for Multiple Sclerosis Therapy

**DOI:** 10.4172/2155-9899.1000488

**Published:** 2017-01-31

**Authors:** Susanta Mondal, Sridevi Dasarathi, Kalipada Pahan

**Affiliations:** Department of Neurological Sciences, Rush University Medical Center, Chicago, IL, USA

**Keywords:** Multiple sclerosis, Experimental allergic encephalomyelitis, Immunomodulation, Glyceryl tribenzoate, TGF-beta, Regulatory T cells

## Abstract

Multiple sclerosis (MS) is the most common autoimmune demyelinating disease of the central nervous system (CNS). Here, we have explored a novel use of glyceryl tribenzoate (GTB), a flavoring ingredient, in ameliorating the disease process of experimental allergic encephalomyelitis (EAE), an animal model of MS, via TGF-β. Oral feeding of GTB suppressed clinical symptoms of adoptively-transferred relapsing-remitting (RR) EAE in recipient mice and suppressed the generation of encephalitogenic T cells in donor mice. GTB also attenuated clinical symptoms of RR-EAE in PLP-TCR transgenic mice and chronic EAE in male C57/BL6 mice. Accordingly, GTB also suppressed perivascular cuffing, preserved the integrity of blood-brain barrier and blood-spinal cord barrier, inhibited inflammation, and stopped demyelination in the CNS of EAE mice. Interestingly, GTB treatment upregulated TGF-β and enriched regulatory T cells (Tregs) in splenocytes as well as *in vivo* in EAE mice. Blocking TGF-β by neutralizing antibodies abrogated GTB-mediated enrichment of Tregs and protection of EAE. These results suggest that oral GTB may be considered as a possible therapy for MS patients.

## Introduction

Tissue-specific autoimmunity can be inhibited by subpopulations of CD4^+^ cells, termed as regulatory T cells (Tregs) [1,2]. These cells are capable of suppressing activation and proliferation of self-reactive T cells and thereby inhibition of immune response of self-reactive T cells against self-antigens [1,2]. A transcription factor of the forkhead/winged-helix family called forkhead box protein 3 (Foxp3) has been implicated in the generation of Tregs. Therefore, the presence of Foxp3 is an important feature of Tregs and these cells often characterized by the presence of Foxp3 and Foxp3^+^ CD4^+^CD25^+^ T cells [1,3]. Under normal physiological conditions, self-reactive T cells are suppressed by Tregs. However, during autoimmune insults, the immune system is altered, resulting in a decrease in the function and the number of Tregs. This dysfunction and/or loss of Tregs ultimately lead to proliferation of self-reactive T cells and subsequent autoimmune attack. Therefore, Treg is increasingly being recognized as an important player in multiple sclerosis (MS) and its animal model experimental autoimmune encephalomyelitis (EAE). A number of articles show that MS is associated with deficiency of Treg numbers and function [4–6]. Accordingly, Tregs participate in the protection and recovery of mice from EAE [7]. According to Venken et al. [8], the expression of Foxp3 and the numbers of peripheral CD4^+^CD25^+^ Foxp3^+^ T cells significantly decrease in relapsing-remitting MS patients compared with those in control subjects. Therefore, upregulation and/or preservation of Tregs may be beneficial for MS.

TGF-β, a multifunctional cytokine, is known to inhibit immune responses is through promoting the generation of Tregs by inducing the expression of Foxp3. Several studies have demonstrated that TGF-β is able to convert CD4^+^CD25^−^ non-Tregs into CD4^+^CD25^+^ Tregs, and this conversion is accompanied with increased expression of Foxp3 [9]. Accordingly, it has been shown that TGF-β is beneficial for EAE and that TGF-β is involved in the recovery from EAE [10]. Therefore, induction of TGF-β could be beneficial and such inducers are poorly understood. Glycerol tribenzoate (GTB) or tribenzoin belonging to the family of benzoic acid is a flavoring ingredient. It is an indirect food additive used as components of adhesives for food packaging, transporting, or holding food. Here, we delineate that GTB treatment was capable of ameliorating the disease process of relapsing-remitting EAE in two different models in female mice and chronic EAE in male mice. Interestingly, GTB enriched Tregs in EAE mice via protection of TGF-β. Abrogation of the GTB-mediated protection from EAE by anti-TGF-β antibody suggests that GTB protects against EAE via TGF-β-Tregs pathway. These results

Suggest that GTB may be used to control autoimmune pathologies in MS via upregulation/maintenance of TGF-β.

## Materials and Methods

### Reagents

Bovine myelin basic protein (MBP), L-glutamine and β-mercaptoethanol were obtained from Invitrogen (Carlsbad, CA). Fetal bovine serum (FBS) and RPMI 1640 were from Mediatech (Washington, DC). Glyceryl tribenzoate, solvent blue 38, cresyl violet acetate, and lithium carbonate were purchased from Sigma Aldrich (St. Louis, MO). Heat-killed *M. tuberculosis* (H37RA) was purchased from Difco Labs. Incomplete Freund’s adjuvant (IFA) was obtained from Calbiochem.

### Induction of EAE

Animal maintaining and experiments were in accordance with National Institute of Health guidelines and were approved by the Institutional Animal Care and Use committee of the Rush University of Medical Center, Chicago, IL.

#### Adoptively-transferred EAE

It was performed as described previously by us [11–15]. Briefly, 4–5 weeks old female SJL/J mice were purchased from Harlan Sprague-Dawley (Indianapolis, IN). Donor mice were immunized s.c. with 400 μg bovine MBP and 60 μg *M. tuberculosis* in IFA [12–15]. Animals were killed 10–12 days postimmunization, and the draining lymph nodes were harvested and single cell suspensions were cultured in RPMI 1640 supplemented with 10% FBS, 50 μg/mL MBP, 50 μM 2-ME, 2 mM L-glutamine, 100 U/mL penicillin, and 100 μg/ml streptomycin. On day 4, cells were harvested and resuspended in HBSS. A total of 2 × 10^7^ viable cells in a volume of 200 μL were injected into the tail vein of naive mice. Pertussis toxin (150 ng/mouse; Sigma-Aldrich) was injected once via i.p. route on 0 day post-transfer (dpt) of cells. Animals were observed daily for clinical symptoms. Six mice were used in each group. Female mice (4–5 week old) were randomly selected for any group. Experimental animals were scored by a masked investigator, as follows: 0, no clinical disease; 0.5, piloerection; 1, tail weakness; 1.5, tail paralysis; 2, hind limb weakness; 3, hind limb paralysis; 3.5, forelimb weakness; 4, forelimb paralysis; 5, moribund or death.

#### Relapsing EAE in 5B6 PLP-TCR Tg mice

PLP_139–151_-specific 5B6 TCR Tg mice were obtained from Prof. Vijay Kuchroo (Harvard Medical School, Boston, MA). Female Tg mice (4–5 weeks old) were immunized with 10 μg of PLP_139–151_ in *M. tuberculosis* in IFA as described [11]. Mice also received pertussis toxin (150 ng/mouse) once on 0 day post-immunization (dpi).

#### Chronic EAE

C57BL/6 mice were immunized with 100 μg of MOG35-55 as described [11]. Mice also received two doses of pertussis toxin (150 ng/mouse) on 0 and 2 dpi.

#### Glyceryl tribenzoate (GTB) treatment

GTB was mixed in 0.5% methylcellulose (MC) and EAE mice were gavaged 100 μL GTB-mixed MC once daily using gavage needle. Therefore, control EAE mice received only MC as vehicle.

#### Histological microscopy

On 14 dpt (first chronic phase), five mice from each of the following groups (control, EAE, EAE+GTB, and EAE+vehicle) were anesthetized. After perfusion with PBS (pH 7.4) and then with 4% (w/v) paraformaldehyde solution in PBS, cerebellum and whole spinal cord was dissected out from each mouse. The tissues were further fixed and then divided into halves: one-half was used for histological staining whereas the other half was used for myelin staining as described earlier [12–15]. For histological analysis, routine histology was performed to obtain perivascular cuffing and morphological details of CNS tissues of EAE mice.

Paraformaldehyde-fixed tissues were embedded in paraffin, and serial sections (4 μm) were cut. Sections were stained with conventional H&E staining method. Digital images were collected under bright-field setting using an ×40 objective. Slides were assessed in a blinded fashion by three examiners for inflammation in different anatomical compartments (meninges and parenchyma). Inflammation was scored using the following scale as described: for meninges and parenchyma: 0, no infiltrating cells; 1, few infiltrating cells; 2, numerous infiltrating cells; and 3, widespread infiltration. For vessels: 0, no cuffed vessel; 1, one or two cuffed vessels per section; 2, three to five cuffed vessels per section and 3, more than five cuffed vessels per section. At least six serial sections of each spinal cord from each of five mice per group were scored and statistically analyzed by ANOVA.

#### Assay of blood-brain barrier (BBB) and blood-spinal cord barrier (BSB) permeabilities

Mice received 200 μl of 20 μM Alexa Fluor 680-SE-NIR (Invitrogen), a near-infrared fluorescent dye, via tail vain. After 2 h, mice were scanned in an Odyssey (ODY-0854; Licor) infrared scanner at 700- and 800-nm channels followed by perfusion with 4% paraformaldehyde. Spinal cord and different regions of brain were scanned in an Odyssey infrared scanner. The red background came from an 800-nm filter, whereas the green signal was from Alexa Fluor 680 dye at the 700-nm channel. The density of the Alexa Fluor 680 signal was quantified with the help of Quantity One version 4.6.2 software using the volume contour tool analysis module.

#### Staining for myelin

Sections were stained with Luxol fast blue for myelin as described earlier [14,15]. Slides were assessed in a blinded fashion for demyelination by three examiners using the following scale: 0, normal white matter; 1, rare foci; 2, a few areas of demyelination; and 3, large areas of demyelination. At least six serial sections of each spinal cord from each of five mice per group were scored and statistically analyzed by ANOVA.

#### Semi-quantitative RT-PCR analysis

Total RNA was isolated from splenic T cells and spinal cord by using the RNeasy mini kit (Qiagen, Valencia, CA) and from spleen and cerebellum by using the Ultraspec-II RNA reagent (Biotecx laboratories, Inc, Houston, TX) following manufacturer’s protocol. To remove any contaminating genomic DNA, total RNA was digested with DNase. Semi-quantitative RT-PCR was carried out as described earlier [14–16] using a RTPCR kit from Clonetech (Mountain View, CA). Briefly, 1 μg of total RNA was reverse transcribed using oligo(dT)12–18 as primer and MMLV reverse transcriptase (Clontech) in a 20 μL reaction mixture. The resulting cDNA was appropriately-diluted, and diluted cDNA was amplified using Titanium Taq DNA polymerase and primers (Table 1). Amplified products were electrophoresed on a 1.8% agarose gels and visualized by ethidium bromide staining. The relative expression of each gene with respect to GAPDH was measured after scanning the bands with a Fluor Chem 8800 Imaging System (Alpha Innotech, San Leandro, CA).

#### Real-time PCR analysis

It was performed using the ABI-Prism7700 sequence detection system (Applied Biosystems, Foster City, CA) as described earlier [14–16]. Briefly, reactions were performed in a 96-well optical reaction plates on cDNA equivalent to 50 ng DNase-digested RNA in a volume of 25 μL, containing 12.5 μL TaqMan Universal Master mix and optimized concentrations of FAM-labeled probe, forward and reverse primers following the manufacturer’s protocol. All primers and FAM-labeled probes for mouse genes and GAPDH were obtained from Applied Biosystems. The mRNA expressions of respective genes were normalized to the level of GAPDH mRNA. Data were processed by the ABI Sequence Detection System 1.6 software and analyzed by ANOVA.

#### TGF-β ELISA

Production of TGF-β in culture supernatant was monitored by ELISA using assay kit from eBioscience (San Diego, CA).

#### Flow cytometry

Two-color flow cytometry was performed as described previously [17,18]. Briefly, 1 × 10^6^ lymph node cells (LNC) or splenocytes suspended in flow staining buffer were incubated at 4°C with appropriately diluted FITC-labeled Ab to CD4 for 30 min, washed, and resuspended in fixation and permeabilization solution. Following incubation in dark for 30 min, cells were washed, blocked with test Fc block (anti-mouse CD16/32) in permeabilization buffer, and subsequently incubated with appropriately diluted PE-labeled Abs to Foxp3 at 4°C in the dark. After incubation, the cell suspension was centrifuged, washed thrice, and resuspended in flow staining buffer. The cells then were analyzed through FACS (BD Biosciences, San Jose, CA). Cells were gated based on morphological characteristics. Apoptotic and necrotic cells were not accepted for FACS analysis.

#### Statistical analysis

All values are expressed as means ± SEM. Differences among means were analyzed by one-way ANOVA or Kruskal-Wallis test (comparison among different groups) and post-hoc pair-wise comparison. In other cases, two sample t tests were also used to compare control *vs.* EAE and EAE *vs.* EAE+GTB treatment.

## Results

### GTB inhibits the adoptive transfer of EAE in female SJL/J mice

**Figure 1:**
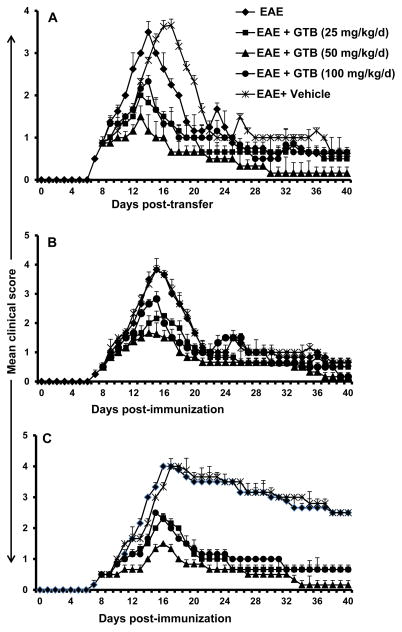
Oral administration of glyceryl tribenzoate (GTB) suppresses clinical symptoms of EAE in adoptive transfer model in female SJL/J mice, female PLP-TCR transgenic (Tg) mice and chronic model in male C57/BL6 mice. **A)** EAE was induced in female SJL/J recipient mice by adoptive transfer of MBP-primed T cells. From 8 dpt, mice were treated with different doses of GTB via gavage. Mice (*n*=6 in each group) were scored daily until 40 dpt. In another group, EAE mice also received methyl cellulose (0.1%) as vehicle. B) PLP-TCR Tg mice were immunized with 10 μg of PLP139–151, and from 8 days post-immunization (dpi) mice were treated with either GTB or vehicle via gavage. Mice (*n*=6 in each group) were scored daily until 40 dpi. C) C57BL/6 mice were immunized with 100 μg of MOG35-55, and from 8 dpi, mice were treated with either GTB or vehicle. Mice (*n*=6 in each group) were scored daily until 40 dpi.

We induced RR-EAE in female SJL/J mice by adoptive transfer of MBP-primed T cells [12,14,15]. These EAE mice were treated with different doses of GTB from 8 days post-transfer (dpt) when these mice exhibited a clinical score of 0.5 or higher. An additional group of mice was treated with vehicle (Figure 1A). Even at a dose of 25 mg/kg body wt/d, GTB significantly inhibited clinical symptoms (Figure 1A). On the other hand, at a dose of 50 mg/kg body wt/d, a dramatic inhibition of clinical symptoms was observed in acute as well as chronic phases of EAE (Table 2 and Figure 1A). Vehicle (0.1% methyl cellulose) remained unable to inhibit the clinical symptoms of EAE (Figure 1A), suggesting the specificity of the effect. However, at a dose of 100 mg/kg body wt/d, GTB was less potent than either 50 mg/kg body wt/d in suppressing clinical symptoms (Figure 1A), suggesting that at higher dose, it may be toxic for EAE mice.

### GTB inhibits clinical symptoms and disease severity of EAE in female PLP-TCR transgenic mice

Next, we examined if GTB treatment was also capable of suppressing the progression of EAE in female PLP-TCR transgenic (Tg) mice. As reported [19], immunization with low dose (10 μg/mouse) of PLP139-151 strongly induced clinical symptoms of EAE in female PLP-TCR Tg mice (Figure 1B). EAE mice were treated with different doses of GTB from 8 days post immunization (dpi). An inhibitory effect of GTB on the clinical symptoms was clearly observed within a few days of treatment (Figure 1B). Greater inhibition was observed on subsequent days of treatment, which was maintained throughout the duration of the experiment (Figure 1B). In this case as well, maximum inhibition of EAE was observed at a dose of 50 mg/kg body wt/d of GTB (Figure 1B). On the other hand, vehicle had no such inhibitory effect (Figure 1B).

#### GTB inhibits chronic EAE in male C57/BL6 mice

While female SJL/J mice are used to induce RR-EAE, chronic form of EAE is modeled in male C57/BL6 mice upon immunization with MOG35-55. Therefore, next, we examined the efficacy of GTB in suppressing the disease process of chronic EAE. Similar to its effect on RR-EAE in female SJL/J mice and PLP-TCR Tg mice, GTB treatment also strongly inhibited the clinical symptoms of EAE in this chronic model (Figure 1C). Again, vehicle had no effect on chronic EAE (Figure 1C), suggesting the specificity of the effect.

### GTB treatment inhibits the generation of encephalitogenic T cells in donor mice

**Figure 2:**
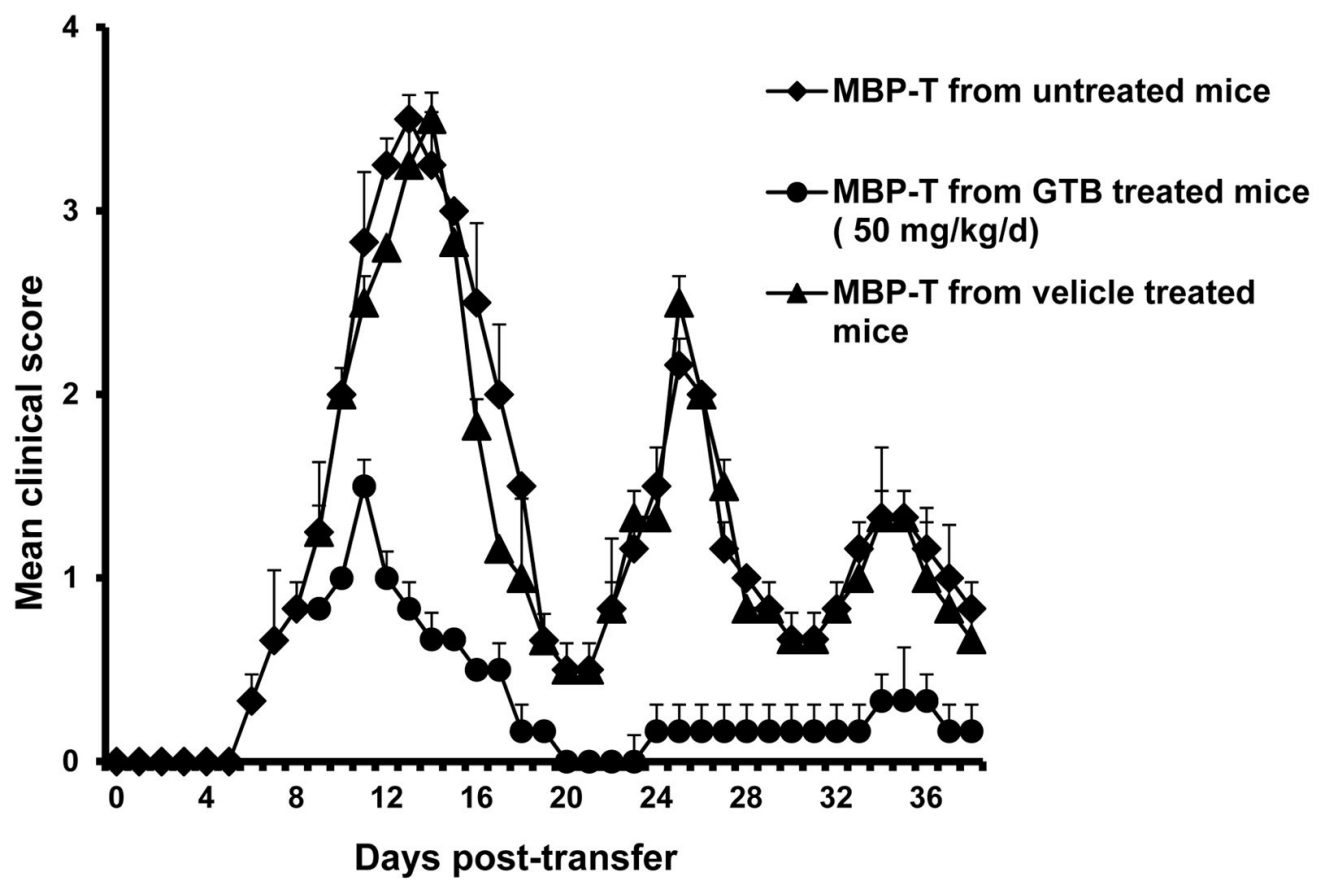
Oral administration of GTB inhibits the generation of encephalitogenic T cells *in vivo* in donor mice. Donor mice (4–6 week old female SJL/J) were immunized with MBP, IFA, and *M. tuberculosis*. From 2^nd^ day of immunization, mice were treated with either GTB (50 mg/kg body wt/d) or vehicle via gavage. On day 12 of immunization, mice were sacrificed, and total LNC were further primed with MBP (50 μg/ml) for 4 days. A total of 2 × 10^7^ viable MBP-primed T cells was adoptively transferred to naïve recipient mice. Six mice (n=6) were used in each group. Mice were examined for clinical symptoms every day until 38 dpt.

T cells isolated from MBP-immunized donor mice are encephalitogenic and adoptive transfer of these T cells induces EAE in recipient mice [12,14,15]. Therefore, we investigated whether treatment of donor mice with GTB was capable of inhibiting the production of encephalitogenic T cells. In order to test this, donor mice were treated with GTB (50 mg/kg body wt/d) orally from the day of MBP immunization. T cells isolated from GTB-treated and untreated MBP-immunized donor mice were then adoptively transferred to recipient mice. Our results showed that mice receiving MBP-primed T cells from GTB-treated donor mice exhibited significantly reduced clinical symptoms and disease severity compared to mice receiving MBP-primed T cells from either untreated donor mice or vehicle-treated donor mice (Figure 2). These results suggest that GTB treatment inhibits the generation of encephalitogenic T cells *in vivo* in donor mice.

### GTB treatment preserves the integrity of blood-brain barrier (BBB) and blood-spinal cord barrier (BSB) in adoptively-transferred EAE mice

**Figure 3:**
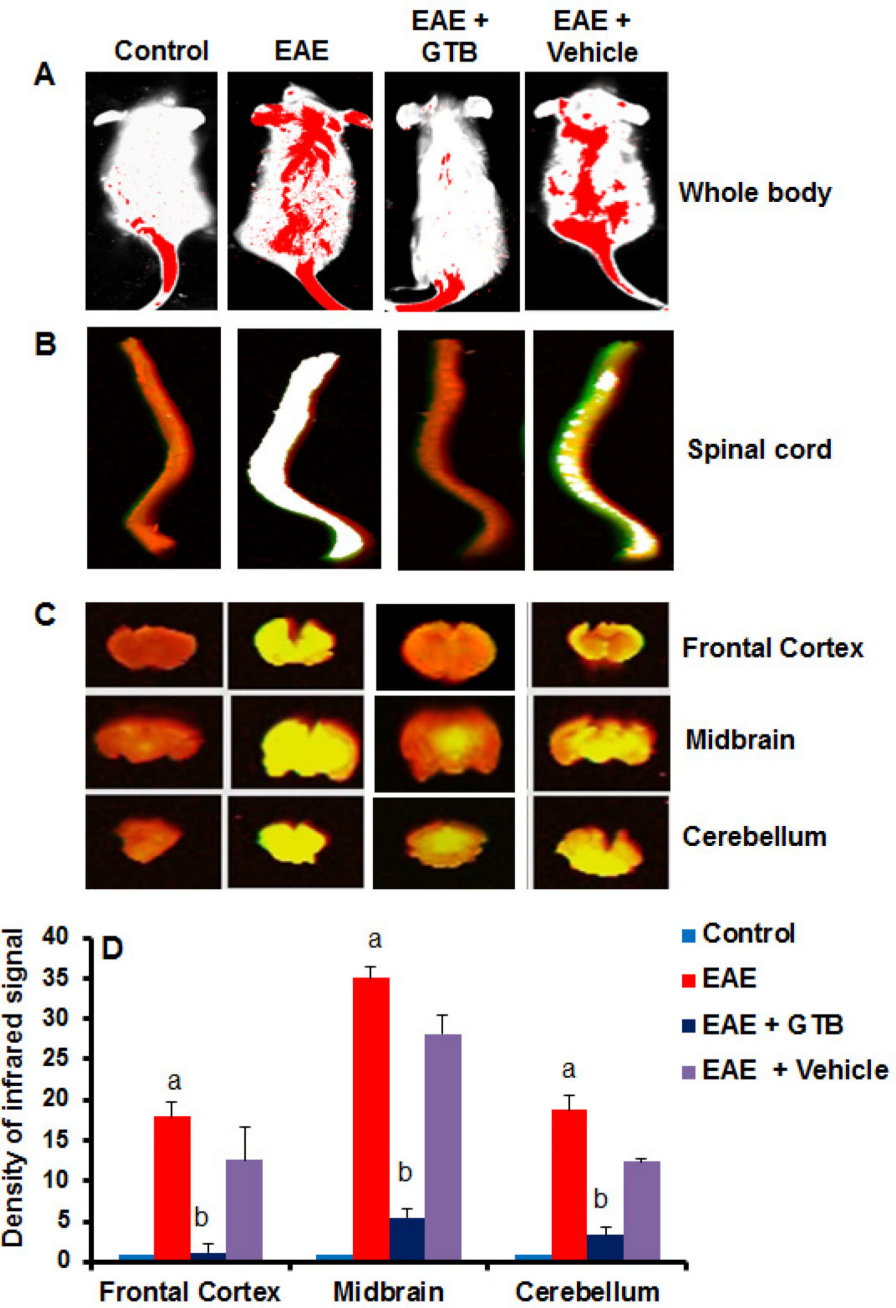
Effect of oral administration of GTB on the integrity of blood-brain barrier (BBB) and blood-spinal cord barrier (BSB) in adoptively-transferred EAE mice. Control, EAE (14 dpt), and either GTB- or vehicle-treated EAE mice (14 dpt receiving GTB/vehicle from 8 dpt) (*n*=5 in each group) received 200 μl of 20 μM Alexa Fluor 680-SE-NIR dye (Invitrogen) via the tail vain. After 4 h, mice were scanned in an Odyssey (ODY-0854; Licor) infrared scanner at the 700- and 800-nm channels. **(A)** Mice were perfused with 4% paraformaldehyde. Spinal cord **(B)** and different parts of the brain **(C)** were scanned in an Odyssey infrared scanner. The red background came from an 800-nm filter, whereas the green signal was from Alexa Fluor 680 dye at the 700-nm channel. The density of the Alexa Fluor 680 signal in different parts of the brain **(D)** was quantified with the help of Quantity One, version 4.6.2 software, using the volume contour tool analysis module. Data are expressed as the mean ± SEM of five different mice; ^a^*p*<0.001 *vs.* normal (HBSS); ^b^*p*<0.001 *vs.* EAE.

BBB and BSB are membranic structures that act primarily to protect the brain and the spinal cord, respectively from chemicals in the blood, while still allowing some essential molecules to enter. It is known that during active MS and EAE, BBB and BSB break down in a section of the brain and spinal cord, respectively due to widespread inflammation thereby allowing different blood molecules and toxins enter into the CNS. Therefore, we investigated if GTB treatment modulated the integrity of BBB and BSB. We injected a near-infrared dye (Alexa Fluor 680-SE-NIR) via tail-vein and 2 h after the injection, live mice were scanned in an Odyssey infra-red scanner. As evidenced from Figure 3A (first lane), infra-red signals were not visible on areas over the brain and the spinal cord in control HBSS-injected mice. On the other hand, in EAE mice, infra-red signals were detected on areas over the brain and the spinal cord (Figure 3A; second column), suggesting possible breakdown of BBB and BSB. However, GTB treatment strongly inhibited the entry of infra-red dye into the CNS of EAE mice (Figure 3A; compare lane 3 with lane 2). In contrast, vehicle treatment did not influence the entry of infra-red dye into the CNS of EAE mice as evidenced by the aligning of infra-red signals over spinal cord and brain (Figure 3A; compare lane 4 with lane 2), suggesting the specificity of the effect.

To confirm these results further, mice were sacrificed, and the spinal cord and different parts of the brain (frontal cortex, midbrain and cerebellum) were scanned for infra-red signals in Odyssey infra-red scanner. Consistent to live mice results, we did not notice much infra-red signal in the spinal cord and different parts of the brain in control HBSS-treated mice (Figures 3B–3D; lane 1) but significant amount of infra-red dye was visible in CNS tissues of EAE mice (Figures 3B–3D; lane 2). Again, treatment of EAE mice by GTB markedly attenuated the entry of infra-red dye into the spinal cord and different parts of the brain (Figures 3B–3D; compare lane 3 with lane 2). Taken together, these results suggest that GTB treatment preserves the integrity of BBB and BSB in EAE mice.

### GTB inhibits infiltration of mononuclear cells, inflammation and demyelination in the spinal cord of EAE

**Figure 4:**
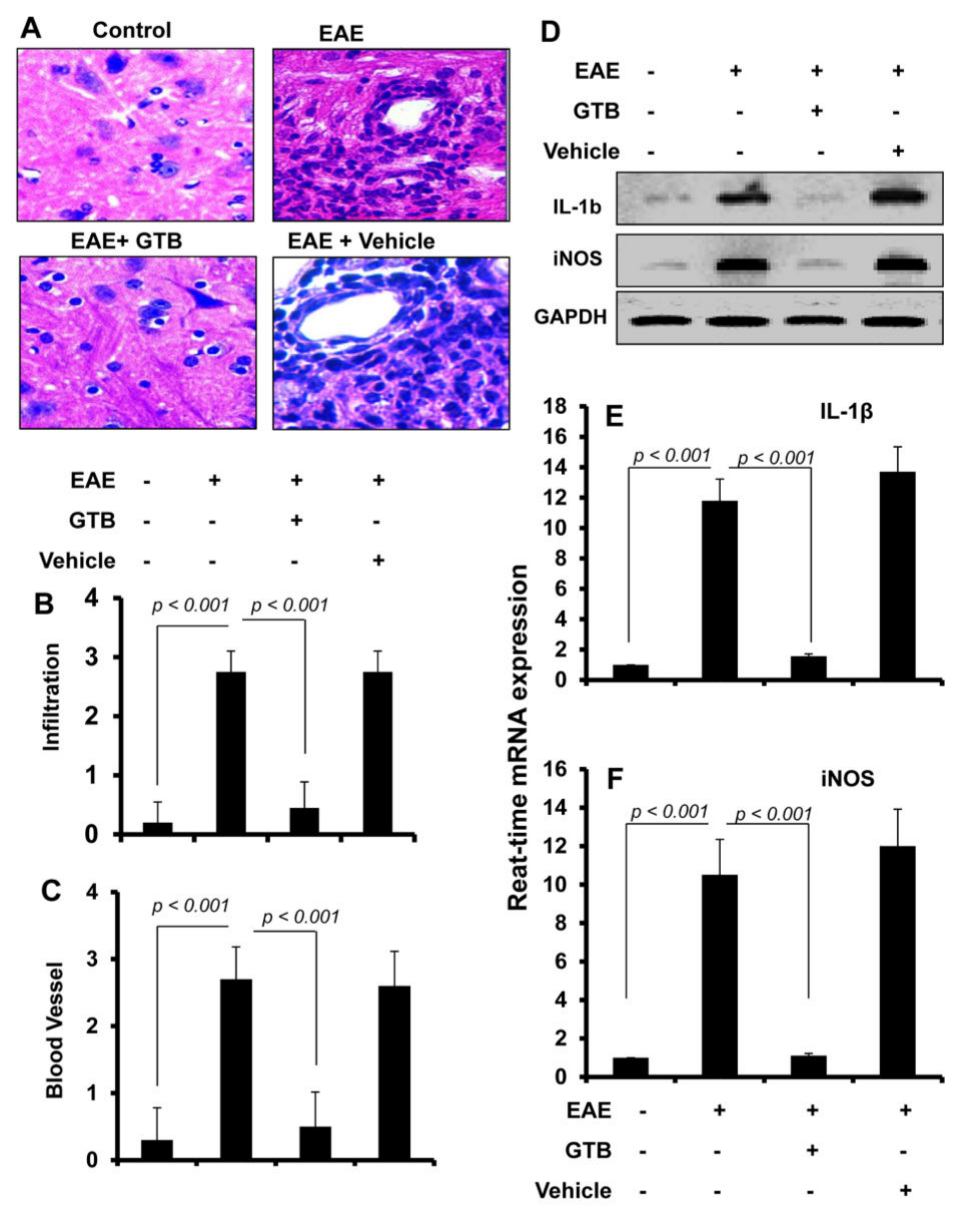
Oral administration of GTB suppresses the infiltration of mononuclear cells in the spinal cord of adoptively-transferred EAE mice. **(A)** Spinal cord sections of control, EAE (14 dpt) and either GTB- or vehicle-treated EAE mice (receiving GTB/vehicle from 8 dpt) were stained with H&E. Digital images were collected under a bright-field setting using a 40X objective. Infiltration **(B)** and cuffed vessel **(C)** were represented quantitatively by using a scale as described by us. Data are expressed as mean ± SEM of five different mice. Spinal cord of normal, EAE, and either GTB- or vehicle-treated EAE mice were analyzed for iNOS and IL-1β mRNAs by semi-quantitative RT-PCR **(D)** and quantitative real-time PCR (E for IL-1β & F for iNOS). Data are expressed as the mean ± SEM of five different mice per group.

Infiltration of autoreactive T cells and associated mononuclear cells is a hallmark of EAE as well as MS [4,20]. We examined whether GTB treatment attenuated infiltration and inflammation in adoptively-transferred EAE mice. Mice receiving GTB from 8 dpt (onset of the acute phase) were sacrificed on 16 dpt. H & E staining showed widespread infiltration of inflammatory cells into the spinal cord (Figure 4A) of EAE mice. On the other hand, GTB treatment markedly inhibited the infiltration of inflammatory cells into the spinal cord of EAE mice (Figure 4A). In contrast, vehicle was unable to inhibit the infiltration of inflammatory cells (Figure 4A). Quantitation of the relative level of inflammatory cells showed that GTB, but not vehicle, dramatically reduced infiltration (Figure 4B) and the appearance of cuffed vessels (Figure 4C) in spinal cord of EAE mice.

Next, we examined whether GTB was capable of inhibiting the expression of proinflammatory molecules in the spinal cord of EAE mice. Marked expression of pro-inflammatory molecules like iNOS and IL-1β was observed in the spinal cord of untreated EAE mice compared to control mice (Figures 4D–4F). However, GTB treatment dramatically reduced the expression of these proinflammatory molecules in the spinal cord of EAE mice (Figures 4D–4F).

**Figure 5:**
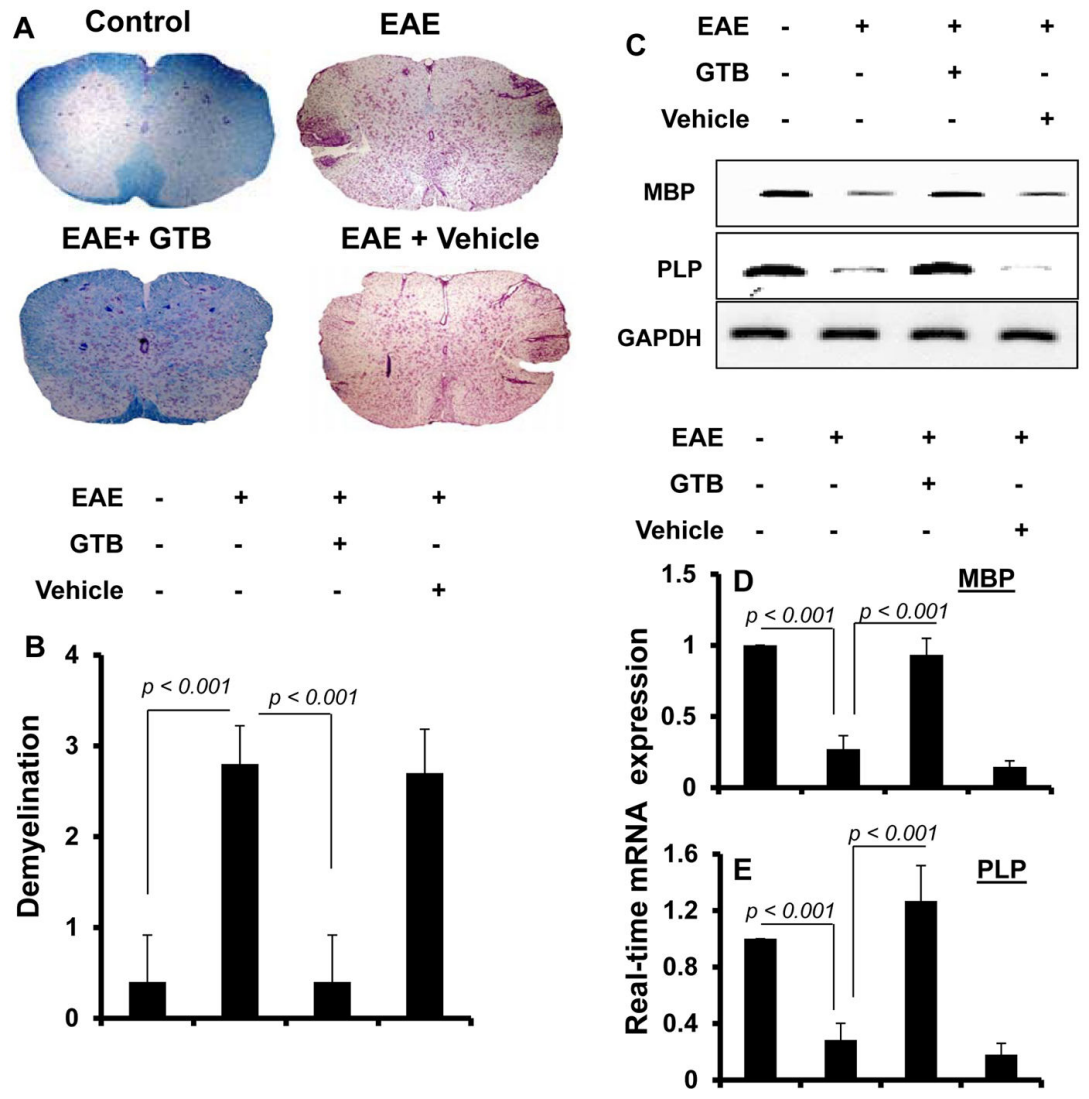
Oral administration of GTB inhibits demyelination in the spinal cord of adoptively-transferred EAE mice. Spinal cord sections of control, EAE (14 dpt) and either GTB- or vehicle-treated EAE mice (receiving GTB/vehicle from 8 dpt) were stained with Luxol fast blue. Digital images were collected under bright field setting using a 40X objective **(A)**. Demyelination was represented quantitatively by using a scale as described by us **(B)**. Data are expressed as the mean ± SEM of five different mice per group. Spinal cord samples were analyzed for MBP and PLP mRNAs by semi-quantitative RT-PCR (C) and real-time PCR (D for MBP & E for PLP). Data are expressed as the mean ± SEM of five different mice per group.

Demyelination is the most important pathological feature in MS, which is also modeled in EAE animals [4,21–23]. Therefore, we examined whether GTB protected EAE mice from demyelination. We stained spinal cord sections by luxol fast blue (LFB) for myelin and observed widespread demyelination zones in the white matter (Figures 5A and 5B). However, GTB treatment remarkably restored myelin level in the spinal cord of EAE mice (Figures 5A and 5B). In contrast, vehicle was unable to restore myelin level in spinal cord of EAE mice (Figures 5A and 5B). To confirm this finding, we monitored the expression of three myelin genes, myelin basic protein (MBP) and proteolipid protein (PLP), and observed a marked loss of mRNA expression of these genes in the spinal cord of untreated EAE mice compared to control mice (Figures 5C–5E). However, significant restoration of myelin gene mRNA expression was observed in the spinal cord of EAE mice that were treated with GTB, but not vehicle (Figures 5C–5E).

### GTB upregulates TGF-β in MBP-primed splenocytes and *in vivo* in EAE mice

**Figure 6:**
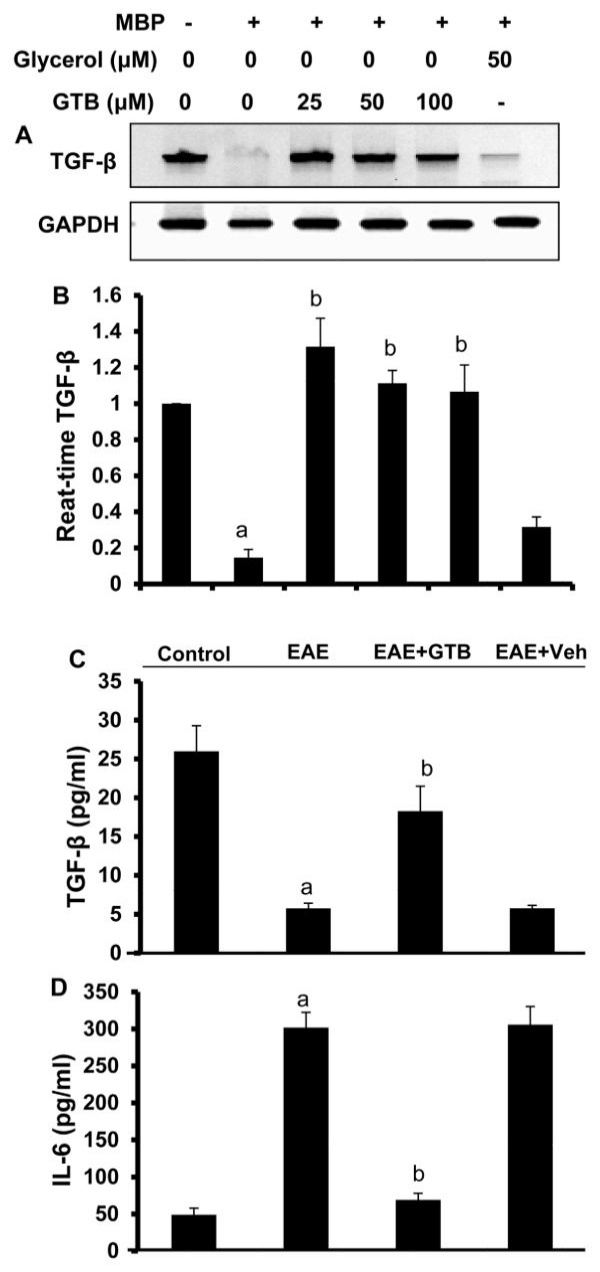
GTB increases the level of TGF-β in MBP-primed splenocytes and in serum of adoptively-transferred EAE mice. Splenocytes isolated from MBP-immunized donor mice were stimulated with MBP in the presence of different concentrations of GTB for 24 h followed by monitoring the mRNA expression of TGF-β by semi-quantitative RT-PCR **(A)** and quantitative real-time PCR **(B)**. Glycerol was run as a negative control for GTB. Data are mean ± SD of three different experiments. ^a^*p*<0.001 *vs.* control; ^b^*p*<0.001 *vs.* MBP. Serum of control, EAE (14 dpt) and either GTB- or vehicle-treated EAE mice (14 dpt receiving GTB/vehicle from 8 dpt) were analyzed for TGF-β (C) and IL-6 (D) by ELISA. Data are mean ± SEM of five different mice per group. ^a^*p*<0.001 *vs.* normal; ^b^*p*<0.001 *vs.* EAE.

Because TGF-β is known to protect mice from EAE, we examined the status of TGF-β after GTB treatment. At first, we monitored TGF-β in GTB-treated MBP-primed splenocytes. Female SJL/J mice were immunized with MBP and on 10 d of immunization, splenocytes were isolated, which were restimulated with MBP in the presence or absence of GTB. As evident from semi-quantitative RTPCR (Figure 6A) and quantitative real-time PCR (Figure 6B), MBP-priming reduced the mRNA expression of TGF-β in splenocytes. However, GTB treatment restored and/or upregulated the mRNA expression of TGF-β in MBP-primed splenocytes (Figures 6A and 6B). These results were specific as glycerol treatment did not exhibit any such effect on TGF-β (Figures 6A and 6B).

Next, we examined the level of TGF-β in serum of GTB-treated and untreated EAE mice by ELISA. EAE mice receiving GTB or vehicle from 8 dpt were sacrificed on 16 dpt followed by analysis of TGF-β in serum. We observed marked decrease in TGF-β in the serum of EAE mice as compared to control mice (Figures 6C). However, treatment of EAE mice with GTB, but not vehicle, protected TGF-β (Figure 6C). While TGF-β alone supports the differentiation of non-Tregs into Tregs, in collaboration with IL-6, TGF-β drives the development of Th17 cells [24], suggesting that IL-6 plays a pivotal role in dictating the balance between the generation of Tregs and Th17 cells. Therefore, we also measured the level of IL-6 in serum of EAE mice. As reported in many instances [25,26], the level of IL-6 was markedly higher in the serum of EAE mice as compared to control mice (Figure 6D). However, in contrast to the modulation of TGF-β, GTB treatment markedly suppressed serum level of IL-6 in EAE mice (Figure 6D).

### Suppression of Th17 response by GTB

**Figure 7:**
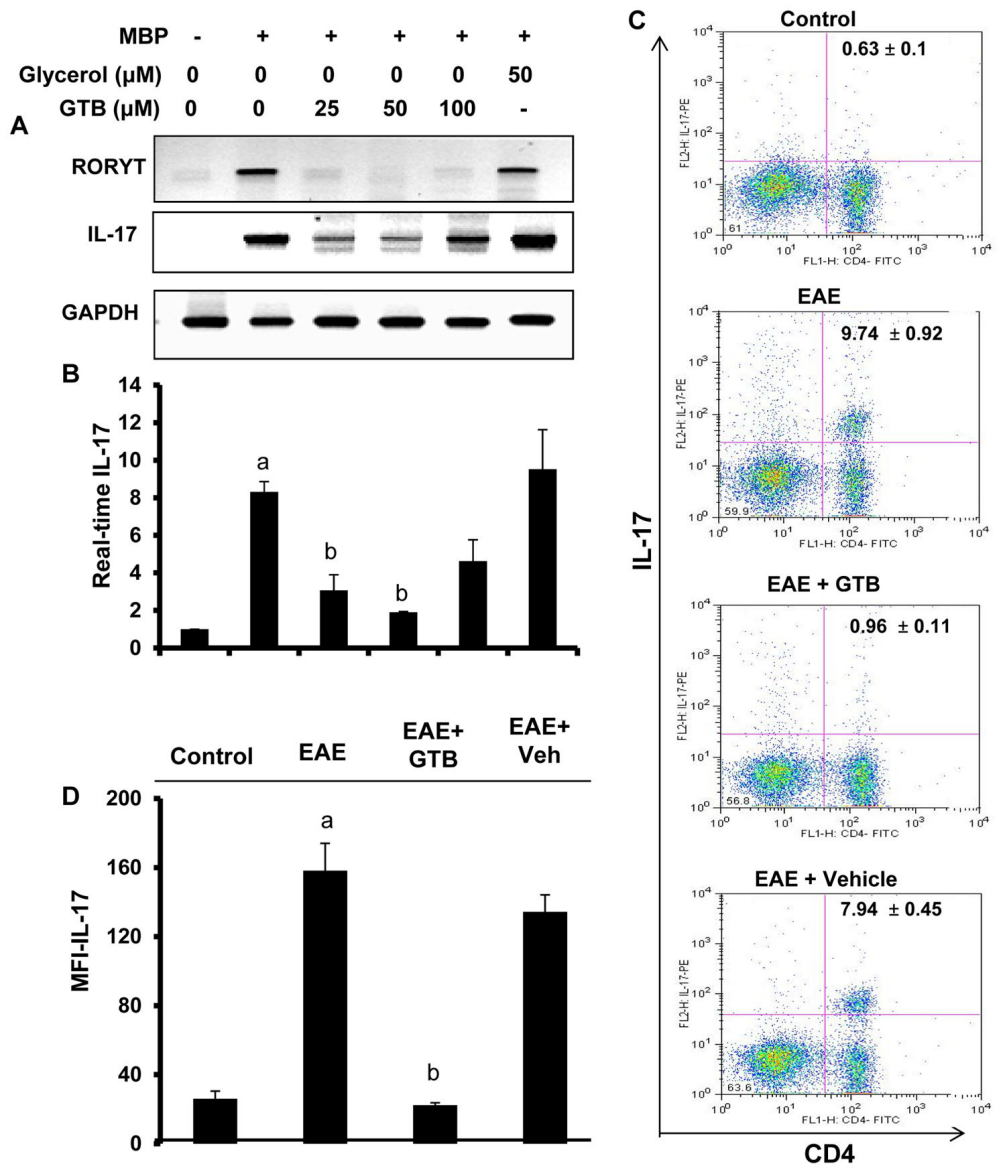
Suppression of Th17 cells by GTB. Splenocytes isolated from MBP-immunized donor mice were stimulated with MBP in the presence of different concentrations of GTB for 24 h followed by monitoring the mRNA expression of RORγt and IL-17 by semi-quantitative RTPCR **(A)** and quantitative real-time PCR **(B)**. Glycerol was run as a negative control for GTB. Data are mean ± SD of three different experiments. ^a^*p*<0.001 *vs.* control; ^b^*p*<0.001 *vs.* MBP. **(C)** Splenocytes isolated from control, EAE (14 dpt) and either GTB- or vehicle-treated EAE mice (14 dpt receiving GTB/vehicle from 8 dpt) were incubated with appropriately diluted PEconjugated anti-IL-17 and FITC-conjugated anti-CD4 Abs followed by FACS analysis. The percentage of cells in the upper right quadrant is indicated. **(D)** The MFI of IL-17 in CD4^+^ population was calculated by using CellQuest software. Data are mean ± SD of three different experiments. ^a^*p*<0.001 *vs.* control; ^b^*p*<0.001 *vs.* MBP.

After the discovery of IL-23, Th17 cells are considered to play a more active role than Th1 cells in the disease process of EAE and MS [27]. Since GTB suppressed the level of IL-6, one of the drivers of Th17, in EAE mice, we examined the effect of GTB on Th17 response. While MBP-priming increased the mRNA expression of IL-17 and RORγt in splenocytes, GTB markedly suppressed the MBP-induced upregulation of IL-17 and RORγt mRNAs (Figures 7A and 7B). Therefore, next, we investigated whether GTB treatment was capable of suppressing Th17 response *in vivo* in EAE mice. EAE mice receiving GTB from 8 dpt were sacrificed on 14 dpt, followed by flow cytometric analysis of splenocytes for IL-17. As expected, induction of EAE markedly increased the levels of CD4^+^IL-17^+^ T cells (Figure 7C). However, treatment of EAE mice with GTB, but not vehicle, led to the suppression of CD4^+^IL-17^+^ T cells (Figure 7C). MFI analysis of IL-17 (Figure 7D) within the CD4^+^ population also supported this finding.

### Enrichment of Treg population by GTB

**Figure 8:**
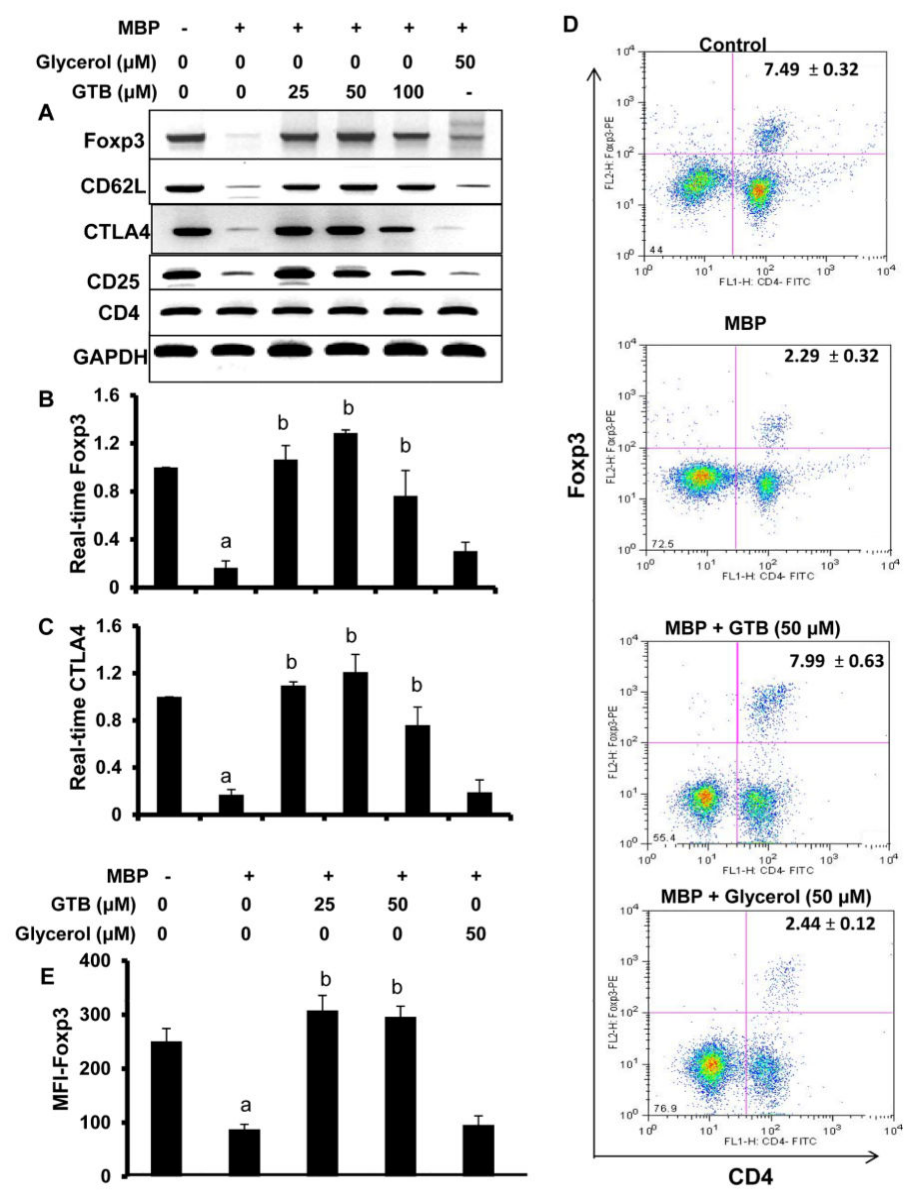
Enrichment of Tregs by GTB in MBP-primed splenocytes. Splenocytes isolated from MBP-immunized donor mice were stimulated with MBP in the presence of different concentrations of GTB for 24 h followed by monitoring the mRNA expression of Foxp3, CD62L, CTLA4, CD4, and CD25 by semi-quantitative RT-PCR **(A)**. The mRNA expression of Foxp3 **(B)** and CTLA4 **(C)** was also checked by quantitative real-time PCR. Glycerol was run as a negative control for GTB. Data are mean ± SD of three different experiments. ^a^*p*<0.001 *vs.* control; ^b^*p*<0.001 *vs.* MBP. **(D)** Splenocytes were incubated with appropriately diluted PE-conjugated anti-Foxp3 and FITC-conjugated anti-CD4 Abs followed by FACS analysis. The percentage of cells in the upper right quadrant is indicated. **(E)** The MFI of Foxp3 in CD4^+^ population was calculated by using CellQuest software. Data are mean ± SD of three different experiments. ^a^*p*<0.001 *vs.* control; ^b^*p*<0.001 *vs.* MBP.

Since TGF-β is known to induce Foxp3^+^ Tregs from CD4^+^CD25^−^ non-Tregs [9] and GTB increases TGF-β production in MBP-primed splenocytes (Figure 6), at first, we examined the effect of GTB on Tregs. Earlier we have demonstrated that antigen priming is capable of suppressing Tregs [18]. Therefore, MBP-primed splenocytes were re-primed with MBP in the presence or absence of different doses of GTB followed by monitoring the expression of the regulatory T cell marker Foxp3. As expected, MBP-priming led to marked loss of Foxp3 (Figures 8A and 8B). However, GTB dose-dependently increased the expression of Foxp3 in MBP-primed splenocytes (Figures 8A and 8B). On the other hand, glycerol had no such on the expression of Foxp3 (Figures 8A and 8B), suggesting the specificity of the effect. Because Foxp3^+^ T cells usually express CD62L, CTLA4 and CD25, we also analyzed the mRNA expression of these molecules. Similar to Foxp3, the mRNA expression of CD62L, CTLA4 and CD25 also decreased upon MBP-priming and GTB rescued the expression of these molecules (Figures 8A–8C). On the other hand, either MBP-priming or GTB treatment had no effect on the mRNA expression of CD4 (Figure 8A), suggesting that these results are not due to any alteration in CD4^+^ T cells. To confirm further, we performed intracellular staining of Foxp3 along with surface staining for CD4. As evident from Figure 8D, MBP-priming reduced Foxp3^+^ T cells. However, treatment of MBP-primed splenocytes with GTB, but not glycerol, led to increase in Foxp3^+^ T cells (Figure 8D). MFI calculation in Figure 8E also shows that GTB treatment resulted in significant increase in Foxp3.

### GTB treatment protects Tregs in EAE mice via TGF-β

**Figure 9:**
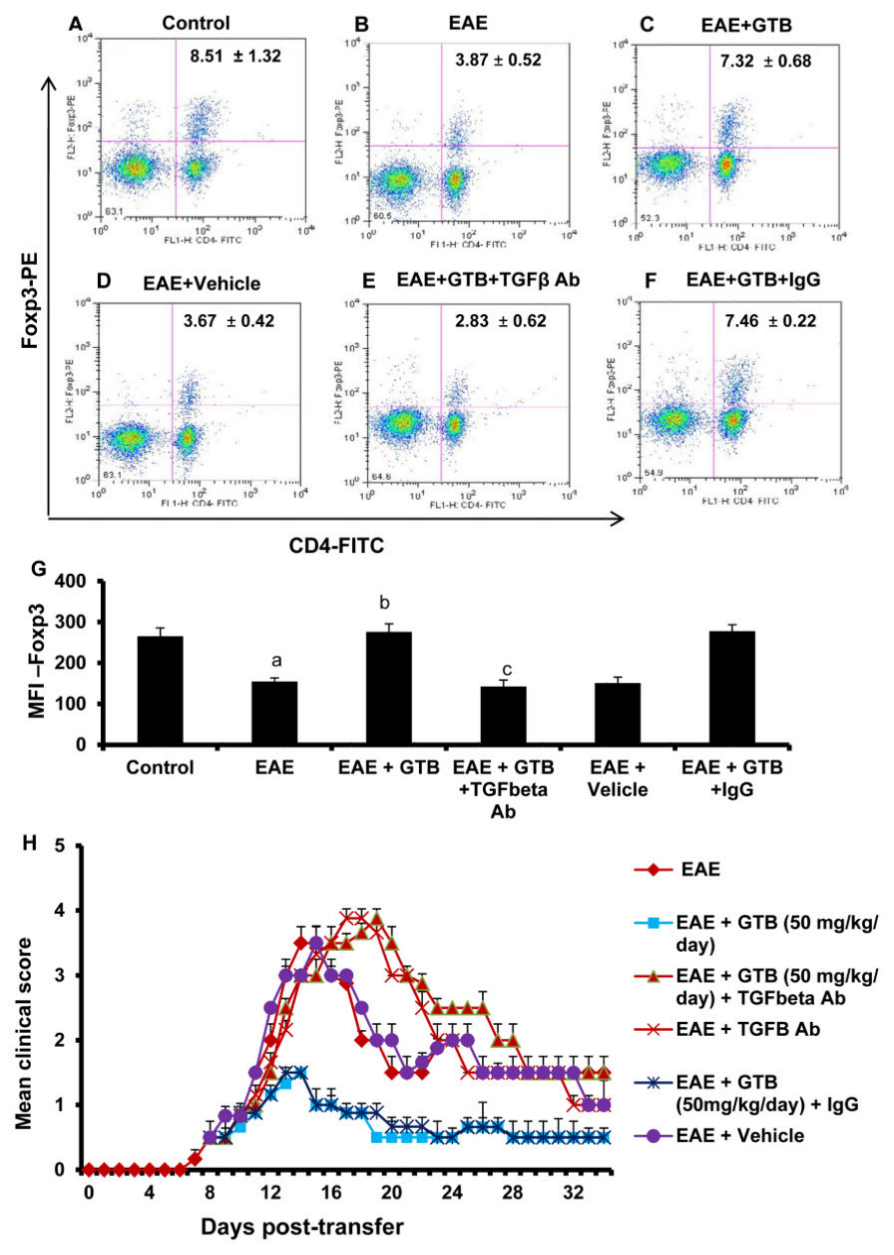
Oral administration of GTB enriches Tregs and protects mice from EAE in adoptive transfer model via TGF-β. EAE was induced in female SJL/J mice by adoptive transfer of MBP-primed T cells and from 8 dpt, mice were treated with GTB (50 mg/kg of body weight/d) via gavage followed by one i.p. injection of anti-TGF-β antibody (30 μg/mouse) on the same day. One group of mice also received same amount of control IgG. LNC isolated from all groups of mice on 14 dpt were analyzed by FACS for Foxp3 and CD4 (**A**, Control; **B**, EAE; **C**, EAE+GTB; **D**, EAE+vehicle; **E**, EAE+GTB+TGF-β Ab; **F**, EAE+GTB+control IgG). (**G)** The MFI of Foxp3 in CD4^+^ population was calculated by using CellQuest software. Data are mean ± SEM of three mice per group. *^a^p*<0.001 *vs.* control; *^b^p*<0.001 *vs.* EAE; *^c^p*<0.001 *vs.* EAE+GTB. Results represent analysis of three mice per group. (**H)** Mice (n=6) were examined for clinical symptoms daily until 38 dpt.

Since GTB treatment upregulates TGF-β in EAE mice, we monitored the status of Tregs in EAE mice. A major population of Tregs is characterized by a transcription factor FoxP3. During autoimmune insults, Tregs become both numerically and functionally defective. Therefore, as expected, we observed significant reduction in Foxp3^+^CD4^+^ population of T cells in EAE splenocytes as evident from FACS dot plot (Figures 9A, 9B and 9G). However, similar to the increase in TGF-β, treatment of EAE mice with GTB, but not vehicle, led to the increase in Foxp3^+^CD4^+^ population in splenocytes (Figures 9A–9D and 9G).

Next, to understand whether GTB enriches Tregs in EAE mice via TGF-β, we employed TGF-β neutralizing antibodies and EAE mice were treated with both GTB and TGF-β neutralizing antibodies. Interestingly, co-treatment of EAE mice with TGF-β neutralizing antibodies abrogated GTB-mediated enrichment and/or protection of Foxp3^+^CD4^+^ population of T cells (Figures 9A–9G). On the other hand, control IgG had no such abrogating effect, suggesting the specificity of the effect (Figures 9A–9G). These results clearly show that GTB treatment protects Tregs in EAE mice via TGF-β.

### GTB suppresses EAE in mice via TGF-β

**Figure 10:**
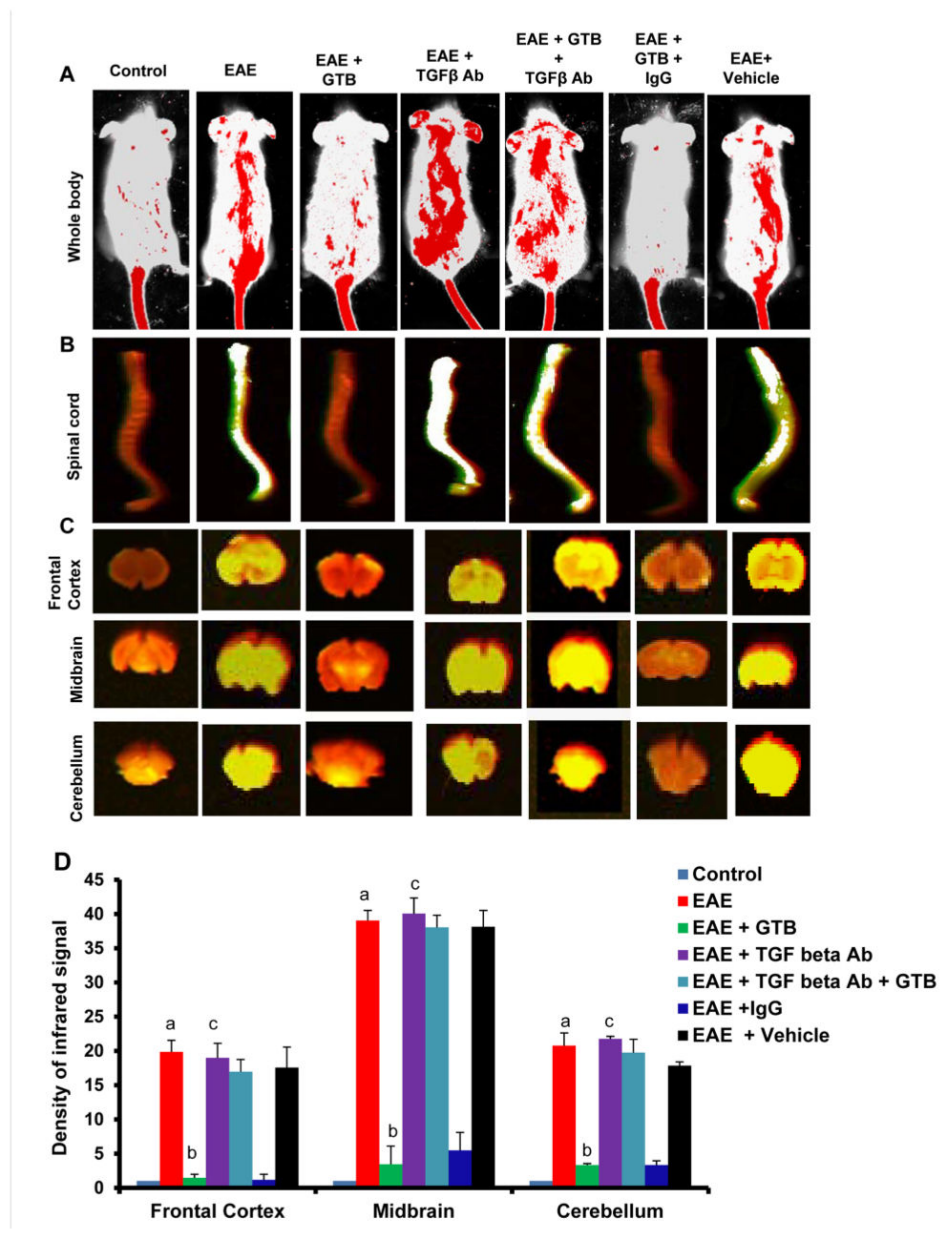
Oral administration of GTB improves the integrity of blood-brain barrier (BBB) and blood-spinal cord barrier (BSB) in adoptively-transferred EAE mice via TGF-β. EAE was induced in female SJL/J mice by adoptive transfer of MBP-primed T cells and from 8 dpt, mice were treated with GTB (50 mg/kg of body weight/d) via gavage followed by one i.p. injection of anti-TGF-β antibody (30 μg/mouse) on the same day. One group of mice also received same amount of control IgG. On 14 dpt, mice received 200 μl of 20 μM Alexa Fluor 680-SE-NIR dye (Invitrogen) via the tail vain. After 4 h, mice were scanned in an Odyssey (ODY-0854; Licor) infrared scanner at the 700- and 800-nm channels **(A)**. Mice were perfused with 4% paraformaldehyde. Spinal cord **(B)** and different parts of the brain **(C)** were scanned in an Odyssey infrared scanner. The density of the Alexa Fluor 680 signal in different parts of the brain **(D)** was quantified with the help of Quantity One, version 4.6.2 software. Data are expressed as the mean ± SEM of three different mice per group. *^a^p*<0.001 *vs.* normal (HBSS); *^b^p*<0.001 *vs.* EAE; *^c^p*<0.001 *vs.* EAE+GTB.

**Figure 11:**
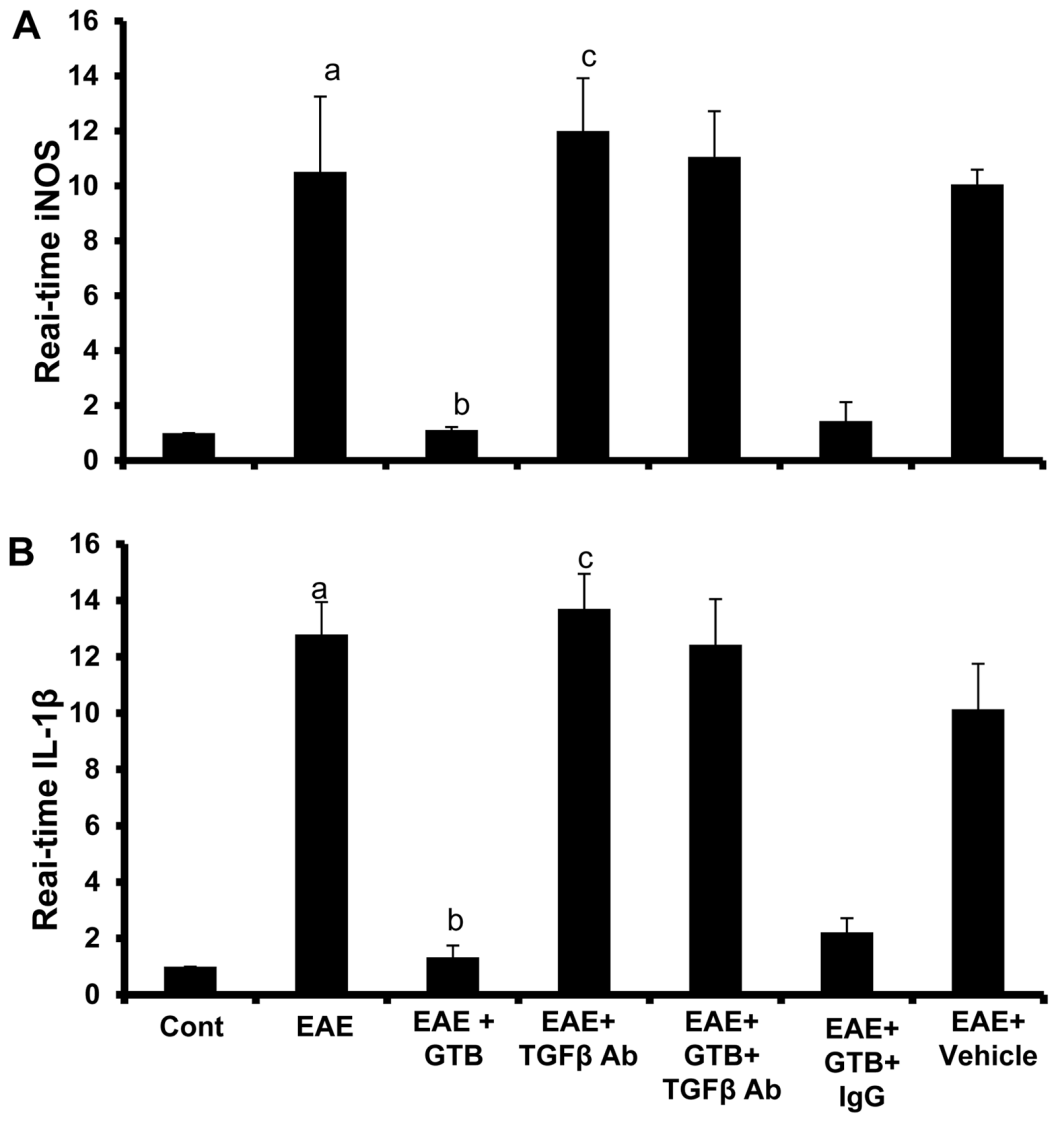
Oral administration of GTB suppresses the expression of proinflammatory molecules in the spinal cord of adoptively-transferred EAE mice via TGF-β. EAE was induced in female SJL/J mice by adoptive transfer of MBP-primed T cells and from 8 dpt, mice were treated with GTB via gavage followed by one i.p. injection of anti-TGF-β antibody on the same day. On 14 dpt, spinal cord of all groups of mice were analyzed for iNOS **(A)** and IL-1β **(B)** mRNAs by quantitative real-time PCR. Data are expressed as the mean ± SEM of three different mice per group. *^a^p*<0.001 *vs.* normal, *^b^p*<0.001 *vs.* EAE; *^c^p*<0.001 *vs.* EAE+GTB.

Next, in order to test the functional significance of GTB-mediated increase in TGF-β further, we examined whether GTB protected mice from clinical symptoms of EAE via TGF-β. Therefore, during GTB treatment, the function of TGF-β was blocked *in vivo* in EAE mice by anti-TGF-β neutralizing antibody. As evident from Figure 9H, GTB treatment ameliorated clinical symptoms of adoptively-transferred RR-EAE. However, functional blocking anti-TGF-β antibody almost completely abrogated the GTB-mediated protective effect on EAE mice (Figure 9H). This result was specific as control IgG had no such effect (Figure 9H). To confirm these results further, we monitored BBB and BSB permeabilities in EAE mice. Anti-TGF-β neutralizing antibody, but not control IgG, markedly abrogated GTB-mediated protection of BBB and BSB integrities in EAE mice (Figures 10A–10D). Accordingly, GTB-mediated suppression of iNOS (Figure 11A) and IL-1β (Figure 11B) in spinal cord of EAE mice was also abolished by anti-TGF-β neutralizing antibody, but not control IgG. Together, these results suggest that GTB suppresses the disease process of EAE via TGF-β.

## Discussion

MS is an inflammatory and degenerative disease of the CNS, leading to debilitating symptoms that vary over time. Current treatments for MS include Tysabri and different forms of interferon-β (IFN-β). However, these drugs have been shown to exhibit reduced effectiveness and severe toxic effects over chronic use. For example, IFN-β has a number of side effects including flu-like symptoms, menstrual disorders in women, decrease in neutrophil and white blood cell count, increase in aspartate aminotransferase and alanine aminotransferase levels, and development of neutralizing antibodies to IFN-β [4,28,29]. Similarly, treatment with Tysabri can cause lung infection, breathing problems, chest pain, wheezing, urinary tract infection, vaginitis, nausea, vomiting, and liver damage. Most importantly, Tysabri also increases the chance of getting progressive multifocal encephalopathy, a severe brain infection, which may ultimately cause disability and death. Therefore, it is essential to describe new, safe and effective therapeutic option for MS.

Glyceryl tribenzoate (GTB) is an FDA-approved indirect food additive that is generally recognized as safe for use in food or food packaging. Here we provide the first evidence that oral administration of GTB is capable of suppressing the disease process of EAE in mice. Our conclusion is based on the following observations. *First*, MBP-primed T cells remained unable to exhibit clinical symptoms of EAE in female SJL/J mice receiving GTB orally. In contrast, MBP-primed T cells induced EAE in SJL/J mice receiving methyl cellulose (vehicle). *Second*, GTB inhibited the generation of encephalitogenic T cells in donor mice. *Third*, GTB treatment preserved the integrity of BBB and BSB in EAE mice. Accordingly, GTB also inhibited the invasion of mononuclear cells into the spinal cord of EAE mice. *Fourth*, therapeutic treatment of EAE animals with GTB inhibited the expression of proinflammatory molecules (iNOS and IL-1β), and restored myelination and the expression of myelin genes within the CNS. We did not notice any side effect (e.g. hair loss, weight loss, diarrhea, untoward infection, etc.) in any of the mice used during five weeks of GTB treatment at doses ranging from 25 to 100 mg/kg body wt/d. These results suggest that GTB may be considered to mitigate the disease process in MS patients.

Decrease in the number of CD4^+^Foxp3^+^ T cells as well as the expression level of Foxp3 is becoming an important characteristic of relapsing-remitting MS and other lymphoproliferative autoimmune disorders [7,8,30]. Therefore, the upregulation of Foxp3^+^ Tregs might be useful for suppressing the activation of autoimmune T cells and controlling autoimmune disorders. Here, we provide the first evidence that GTB treatment enriches Foxp3^+^ Tregs in MBP-primed splenocytes and that oral feeding of GTB protects Tregs *in vivo* in mice. According to our earlier finding [18], MBP-priming reduced the expression of Foxp3 in splenocytes. However, GTB treatment upregulated the expression of Foxp3 in MBP-primed splenocytes. This was also verified by dual-label (CD4 and Foxp3) FACS analysis of splenocytes. Moreover, Foxp3^+^ Tregs are also identified by CD25, CD62L, CTLA4, etc. Accordingly, we have found decreased expression of CD25, CD62L and CTLA4 in MBP-primed splenocytes as compared to normal splenocytes. Again, GTB treatment abrogated the loss of CD25, CD62L and CTLA4 in MBP-primed splenocytes. These results were specific as neither MBP-priming nor GTB treatment had any effect on CD4, suggesting that upregulation of Foxp3 and Treg markers by GTB is not due to any reduction of CD4^+^ cells. Consistent to the fact that Tregs release IL-35 for controlling the proliferation of Th17 cells [31], GTB suppressed the expression of IL-17 and reduced the population of CD4^+^IL-17^+^ T cells in MBP-primed splenocytes. Accordingly, induction of EAE reduced the population of CD4^+^Foxp3^+^ T cells while increasing the population of CD4^+^IL-17^+^ T cells. However, GTB treatment increased the population of CD4^+^Foxp3^+^ T cells and inhibited the Th17 immune response in EAE mice.

How does GTB induce Tregs? TGF-β is an important inducer of Tregs as TGF-β is capable of swiching non-Tregs to Foxp3^+^ Tregs [9]. TGF-β is known to induce the phosphorylation of Smad3 and Smad4 and the resulting P-Smad3/P-Smad4 complex then binds to the Foxp3 gene promoter to drive its transcription [32,33]. Therefore, we hypothesized that GTB treatment could enrich Tregs via upregulation of TGF-β. Accordingly, GTB increased the expression of TGF-β in MBP-primed splenocytes. Upregulation of TGF-β in serum of GTB-treated EAE mice as compared to vehicle treatment and abrogation of GTB-mediated protection of Foxp3 by anti-TGF-β neutralizing antibody suggest that GTB treatment protects Tregs via TGF-β production. Similarly, anti-TGF-β antibody also neutralized the protective effect of GTB against EAE, indicating that the effect of GTB is TGF-β-dependent. While TGF-β alone promotes Tregs, in the presence of IL-6, TGF-β drives Th17 cells [24]. Accordingly, GTB treatment suppressed the production of IL-6 in the serum of EAE mice, indicating the specificity of the effect.

In summary, we have demonstrated that oral administration of GTB attenuates the disease process of EAE via TGF-β-mediated upregulation of Tregs. These results highlight a novel immunomodulatory role of GTB and suggest that this indirect food additive may be explored for therapeutic intervention in MS and other demyelinating disorders.

## Figures and Tables

**Table 1:** Sequences of primers used in this study.

TGF-β	Sense: 5′-GCCGCTTCTGCTCCCACTCC -3′ Antisense: 5′-TGGGGGTCAGCAGCCGGTTA -3′
IL-17	Sense: 5′-GCTGACCCCTAAGAAACCCC -3′ Antisense: 5′-GAAGCAGTTTGGGACCCCTT -3′
RORγt:	Sense: 5′-ACGCGTCAGCAGAACTGCCC -3′ Antisense: 5′-GGAGGCCCCCTGGACCTCTG -3′
Foxp3	Sense: 5′-CAGCTGCCTACAGTGCCCCTAG -3′ Antisense: 5′-CAT TTG CCA GCA GTG GGT AG -3′
CD62L	Sense: 5′-AGC CTC TTG CCA GCC AGG GT -3′ Antisense: 5′-CCA GCC CCG AGA ATG CGG TG -3′
CTLA4	Sense: 5′-GGT CCG GGT GAC TGT GCT GC -3′ Antisense: 5′-CCC GTT GCC CAT GCC CAC AA -3′
CD25	Sense: 5′-AGC CAA GTA GGG TGT CTC TCA ACC -3′ Antisense: 5′-GCC CAG GAT ACA CAG TGA AGA ACG -3′
CD4	Sense: 5′-CCA ACA AGA GCT CAA GGA GAC CAC -3′ Antisense: 5′-CGT ACC CTC TTT CCT AGC AAA GGA -3′
iNOS	Sense: 5′-CCCTTCCGAAGTTTCTGGCAGCAGC-3′ Antisense: 5′-GGCTGTCAGAGCCTCGTGGCTTTGG3
IL-1β	Sense: 5′-CTCCATGAGCTTTGTACAAGG-3′ Antisense: 5′-TGCTGATGTACCAGTTGGGG-3′
MBP	Sense: 5′-TGGAGAGATTCACCGAGGAGA-3′ Antisense: 5′-TGAAGCTCGTCGGACTCTGAG-3′
PLP	Sense: 5′-CTTTGCTTCCCTGGTGGCCA-3′ Antisense: 5′-TGTTGGCCTCTGGAACCCCT-3′
GAPDH	Sense: 5′-GGTGAAGGTCGGTGTGAACG3′ Antisense: 5′-TTGGCTCCACCCTTCAAGTG-3′

**Table 2:** Effect of GTB treatment on clinical symptoms of RR-EAE.

Treatment	Incidence	Mean Peak Clinical Score	Suppression of EAE (%)	
			Incidence	Score
No treatment	6/6	3.5		
GTB (50 mg/kg body wt/d)	2/6	1.5	66.67^a^	57^a^
Vehicle (100 μl/mouse/d) from 8 dpt	6/6	3.66	0	0

EAE was induced in female SJL/J mice through adoptive transfer of MBP-primed T cells. Groups of donor mice receiving MBP-primed T cells were treated with either GTB or vehicle from 8 dpt. A clinical score of 2 was considered as the incidence of EAE in mice.

a *p*<0.001.

## Abbreviations

GTB: Glyceryl Tribenzoate
EAE: Experimental Allergic Encephalomyelitis
RR: Relapsing Remitting
Tregs: Regulatory T cells
TGF-β: Transforming Growth Factor-β
Foxp3: Forkhead box protein 3
PLP: Proteolipid Protein
MBP: Myelin Basic Protein
IL-17: Interleukin-17
Th17: T helper cell 17
BBB: Blood-brain Barrier
BSB: Blood-spinal cord Barrier
